# Lunar synchronization of hemostasis and immunity validates prophetic timing of hijama therapy: A multicenter study from Yemen

**DOI:** 10.1016/j.jtumed.2025.12.004

**Published:** 2026-01-06

**Authors:** Naif T. Ali, Radfan S. Abdullah, Ibrahim Ahmed, Othman Mohammed, Akram Jamal, Fawaz Ahmed, Bushra Dahan, Alawy Adel, Tofaha Abdullah, Monther Saleh, Shadia Hamid, Naif Mohammed, Khawla Mohammed, Yaqoub Khalid, Sabran Nabil, Abdulmajeed Abdualkareem

**Affiliations:** a Department of Laboratory Sciences, Radfan University College, Lahej University, Yemen; b Department of Health Sciences, Faculty of Medicine and Health Sciences, University of Sciences and Technology, Aden, Yemen; c Department of Laboratory Sciences, Aden Gulf International University Yemen

**Keywords:** البيولوجيا القمرية, الحجامة, الإرقاء, التخثّر, الوظيفة المناعية, الطب النبوي, Coagulation, Hemostasis, Hijama, Immune function, Lunar biology, Prophetic medicine

## Abstract

**Objective:**

This study investigated lunar-phase-dependent variations in hematological and immune parameters to empirically validate traditional Islamic recommendations for performing hijama (wet cupping) during specific mid-Hijri lunar days (17th–21st).

**Methods:**

A total of 258 healthy adults (200 men and 58 women) from Ad'Dla Governorate underwent morning venipuncture (0700–0900, ≥8 h fasting) during the new moon (1st–5th) and full moon (17th–21st) phases. Complete blood counts and coagulation profiles were analyzed with automated systems. Hydration was standardized (500 ml water 1 h pre-sampling), and laboratory technicians were blinded to phase assignments. Statistical analysis included paired t-tests and ANOVA with α = 0.05.

**Results:**

Platelets increased by 5.9% in men (276.67→292.88 × 10^9^/L, p = 0.002) and 2.7% in women (304.79→313.10 × 10^9^/L, p = 0.012) mid-month. Clotting time decreased by 33.3% (6.0→4.0 min, p < 0.001). Monocytes increased 106% in young men (p < 0.001). All changes remained within clinical ranges but showed large effect sizes (Cohen's d = 0.42–0.82).

**Conclusion:**

Mid-Hijri month coincided with enhanced hemostatic and immune activity, thereby validating prophetic hijama timing. The findings established a physiological framework for lunar chronotherapy, although their generalizability requires validation in diverse populations.

## الملخص

**أهداف البحث:**

هدفت هذه الدراسة إلى استقصاء التغيّرات المعتمدة على أطوار القمر في المؤشرات الدموية والمعايير المناعية، بهدف التحقق تجريبيًا من التوصيات الإسلامية التقليدية التي تشير إلى أفضلية إجراء الحجامة خلال أيام منتصف الشهر الهجري (21–17).

**طرق البحث:**

شملت العينة 258 بالغًا أصحاء (200 رجل و58 امرأة) من محافظة العُديلة، وخضع المشاركون لسحب دم وريدي صباحي (07:00–09:00، صيام ≤ 8 ساعات) خلال مرحلتي المحاق (1–5) والبدر (17–21). تم تحليل تعداد الدم الكامل ومؤشرات التخثّر باستخدام أنظمة آلية ذاتية التشغيل. وجرى توحيد الإماهة عبر تناول 500 مل ماء قبل ساعة من السحب، كما تم تعمية الفنيين عن الطور القمري لكل عينة. شمل التحليل الإحصائي اختبار تي للعينات المزدوجة وتحليل التباين عند مستوى دلالة 0.05.

**النتائج:**

ارتفع عدد الصفائح الدموية بنسبة 5.9% لدى الرجال (276.67→292.88 ×10^9^/لتر، p = 0.002) وبنسبة 2.7% لدى النساء (304.79→313.10 ×10^9^/لتر، p = 0.012) خلال منتصف الشهر. كما انخفض زمن التخثّر بنسبة 33.3% (6.0→4.0 دقائق، p < 0.001). وأظهرت الخلايا الوحيدة زيادة قدرها 106% لدى الرجال اليافعين (p < 0.001). وعلى الرغم من بقاء التغيرات ضمن الحدود السريرية الطبيعية، إلا أنها ارتبطت بأحجام تأثير كبيرة (Cohen’s d = 0.42–0.82).

**الاستنتاجات:**

يرتبط منتصف الشهر الهجري بزيادة ملحوظة في نشاط الجهازين الدموي والمناعي، مما يدعم توقيت الحجامة المنسوب إلى السنة النبوية. وتوفر هذه النتائج أساسًا فسيولوجيًا لمفهوم "العلاج الزمني القمري"، إلا أن تعميمها يستلزم التحقق في مجموعات سكانية مختلفة.

## Introduction

Biological rhythms govern nearly every physiological process in the human body, including hormone secretion and immune activation. Whereas circadian rhythms driven by solar light are well characterized, the influence of lunar cycles, particularly the moon's gravitational and photic effects, has received less attention in clinical research. Nonetheless, the moon's role in orchestrating tides, animal behavior, and human sleep patterns is well documented.^1^^,^^2^

Human blood, composed of approximately 55% plasma, exhibits non-Newtonian fluid properties that may respond to subtle gravitational variations.^2^ Contemporary studies suggest that lunar cycles modulate platelet activity through 12.4-h ultradian rhythms,^3^ whereas microgravity research has demonstrated gravity's role in hematopoiesis.^4^^,^^5^ These findings provide a mechanistic foundation for investigating lunar hematological influences.

In traditional systems of medicine, such as traditional Chinese medicine, Ayurveda, and Islamic prophetic medicine (Tibb al-Nabawi), lunar phases have long been recognized to influence the timing of certain therapeutic interventions. Hijama (wet cupping) is recommended in prophetic traditions to be performed on the 17th, 19th, or 21st days of the lunar (Hijri) month, coinciding with the full moon. These recommendations, found in classical texts such as Tibb al-Nabawi by Ibn Qayyim, assert that this timing enhances detoxification and physiological balance.^6^^,^^7^

The Prophet Muhammad (PBUH) specifically endorsed hijama on the 17th, 19th, and 21st lunar days,^8^ coinciding with peak gravitational influence during full moon. Classical scholar Ibn Qayyim historically described this connection, noting that blood has its tides (Tibb al-Nabawi), a foundational principle now being examined through modern hematology.

Despite its historical foundation, the empirical basis for lunar-timed hijama remains underexplored. Most prior studies on cupping therapy have focused on clinical outcomes,^9^^,^^10^ without examining whether the timing within the lunar month might modulate hematological or immunological parameters. Additionally, although some investigations have documented lunar influences on sleep,^1^ behavior,^11^ and mood disorders,^12^ studies linking lunar phase to hematology or coagulation dynamics have been sparse and often contradictory. For example, one study^13^ observed platelet changes post-hijama but did not perform a comparison across lunar phases or control for confounders. Importantly, conflicting evidence exists across research domains. Another study^14^ has found no lunar influence on spontaneous deliveries, thus underscoring the methodological heterogeneity in lunar biology studies.

Given the blood-based nature of hijama, understanding whether physiological shifts occur during specific lunar phases has potential scientific and clinical importance. Modern chronobiology, including research on microgravity and gravitational biology,^4^^,^^5^ supports the plausibility that subtle environmental forces, including lunar tides, might influence hematopoiesis, immune regulation, and vascular tone. We propose integrated chronobiological synchronization, wherein lunar photic and gravitational cues collectively entrain hematological rhythms, rather than attributing the effects solely to weak gravitational forces.

This study addresses a critical gap by evaluating whether key hematological and coagulation parameters vary across two key lunar windows: early Hijri month (1st–5th) and mid-Hijri month (17th–21st), encompassing the traditionally recommended hijama dates. To our knowledge, this study is the first to quantify lunar-synchronized physiological changes in a healthy human cohort while controlling for fasting, hydration, activity, and diurnal variation.

This study was aimed at determining whether hematological and coagulation parameters, including platelet count, clotting time, and immune differentials, might exhibit significant variation between early and mid-lunar phases in the Hijri calendar. Consequently, we sought to empirically validate traditional recommendations for hijama timing and contribute to the growing field of lunar chronotherapy.

## Materials and Methods

### Study design, setting, and participants

A multicenter, cross-sectional study was conducted in Ad'Dla Governorate, Yemen, during two defined lunar phases: early Hijri month (1st–5th) and mid-Hijri month (17th–21st), corresponding to traditional recommendations for hijama and verified via official lunar calendars from the Yemeni Ministry of Religious Affairs. The study received ethical approval from the Ad'Dla Medical Research Ethics Committee (AMREC-2024-087) and adhered to STROBE guidelines. A total of 258 healthy adult volunteers were recruited from the community. Stratified sampling was used to ensure representation across sex and age groups. The inclusion criteria were adults 18–50 years of age with no current illnesses, known hematologic/coagulation disorders, use of medications affecting blood/immune parameters, history of hijama, and blood donation or blood transfusion in the prior 3 months. Exclusion criteria included pregnancy, fever or infection within 2 weeks, use of NSAIDs, anticoagulants, or corticosteroids, and being shift workers or having irregular sleep cycles.

### Standardization protocols, blood collection, and Laboratory Analysis

To minimize confounding variables, all participants adhered to a strict pre-sampling protocol involving an overnight fast of ≥8 h and refrained from fluid intake for ≥2 h. Blood collection was fixed between 0700 and 0900 AM to control for diurnal variation, and physical exertion was prohibited within the preceding hour. To standardize hydration, participants consumed 500 ml water 1 h before phlebotomy, and euhydration was confirmed via visual assessment of urine color (light yellow). Ambient temperature and humidity were monitored to account for environmental influences, and all laboratory analyses were conducted in a temperature-controlled environment (22 ± 1 °C).

Trained phlebotomists performed venipuncture, collecting 3 ml blood into K_2_EDTA tubes for complete blood count (CBC) determination and 2 ml into 3.2% sodium citrate tubes for coagulation assays. To ensure blinding, we anonymized all samples with codes. Laboratory technicians were unaware of the lunar phase assignments. Samples from both phases were analyzed in randomized sequences across multiple days with the same equipment, to eliminate batch effects, and all assays were completed within 2 h of collection. Automated analyzers were used to minimize operator-dependent variability: CBC was performed on a Sysmex XP-300 analyzer (Sysmex Corp., Japan), and coagulation profiles (prothrombin time [PT] and activated partial thromboplastin time [APTT]) were assessed with an STA Compact system (Stago, France). Clotting time (CT) and bleeding time were measured via standardized capillary methods.

### Quality control and statistical analysis

Rigorous quality control was maintained through daily calibration of analyzers with NIST-traceable controls. Internal quality control samples were run concurrently with participant samples, and the inter-assay coefficients of variation remained below 3% for CBC and below 5% for coagulation assays.

Statistical analyses were performed in SPSS v23 (IBM, USA). After normality assessment with the Shapiro–Wilk test, parametric tests (paired t-tests, ANOVA) or non-parametric equivalents (Wilcoxon signed-rank test) were applied, as appropriate. Effect sizes (Cohen's d) were calculated, and adjustment for multiple comparisons was performed with Bonferroni correction (adjusted α = 0.025). Bayesian t-tests were used for key outcomes and provided very strong evidence of lunar-phase effects (BF_10_ > 10 for platelet and CT changes). To further validate robustness, we conducted bootstrapping with 10,000 iterations to confirm 95% confidence intervals for key outcomes.

## Results

### Participant demographics

A total of 258 healthy adults participated in the study (men: n = 200; women: n = 58). Age groups were distributed as follows: young men (18–29 years): 120 participants; older men (30–50 years): 80 participants; and women (18–50 years): 58 participants. All participants completed both early and mid-Hijri phase blood collections. No adverse events were reported. The mean age was 31.2 ± 9.5 years. Baseline hematological and coagulation profiles were within standard clinical ranges. Environmental factors were controlled through standardized sampling conditions. Although the ambient temperature averaged 30 °C, its potential effect was mitigated by indoor climate control (22 ± 1 °C).

### Hematological variation across lunar phases

Significant lunar-phase-dependent changes were observed in platelet counts, white blood cell differentials, and coagulation parameters. Table 1 summarizes key coagulation changes between the new moon and full moon phases.

**Table 1:** Coagulation profile changes across lunar phases.

Parameter	New Moon (Mean ± SD)	Full Moon (Mean ± SD)	% Change	P-value
CT (min)	6.0 ± 2.0	4.0 ± 1.0	−33.3%	<0.001
BT (min)	1.0 ± 0.34	1.0 ± 0.36	NS	0.45
PT (sec)	14.0 ± 1.0	17.0 ± 4.0	+21.4%	<0.01
APTT (sec)	34.0 ± 5.0	42.0 ± 10.0	+23.5%	<0.001

CT, clotting time; BT, bleeding time; PT, prothrombin time; APTT, activated partial thromboplastin time; SD, standard deviation; NS, not significant.

Data are presented as mean ± standard deviation. P-values were derived from paired t-tests. NS denotes a non-significant change (p > 0.05).

**Figure 1:**
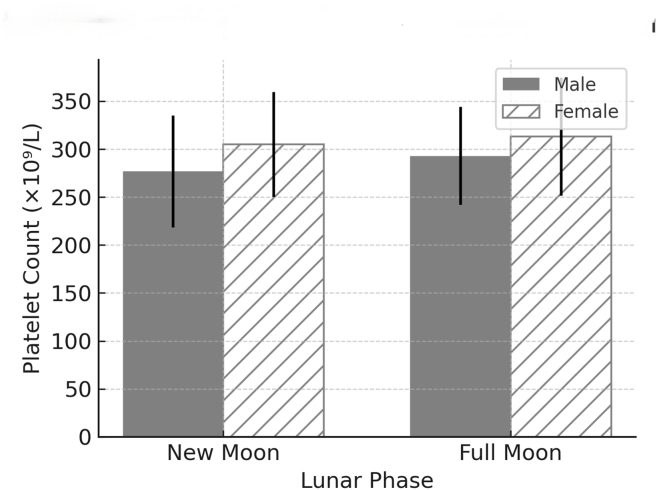
**Platelet dynamics by lunar phase in a Yemeni cohort.** Bar graph illustrating mean platelet counts (± standard deviation) for male and female participants during the new moon (1st–5th Hijri) and full moon (17th–21st Hijri) phases. Platelet counts were significantly elevated during the full moon phase in both sexes (∗p < 0.05, ∗∗p < 0.01, on the basis of paired t-tests).

Although statistically significant, all observed changes remained within standard clinical ranges. For example, platelet counts significantly increased during the full moon phase in both men (+5.9%, p = 0.002) and women (+2.7%, p = 0.012) but remained within the normal range of 150–400 × 10^9^/L. Similarly, the observed APTT prolongation (23.5%) remained below therapeutic anticoagulation thresholds, and the monocyte elevation (106%) was transient and asymptomatic. The platelet dynamics are visually demonstrated in Figure 1.

### Immune cell variations

**Figure 2:**
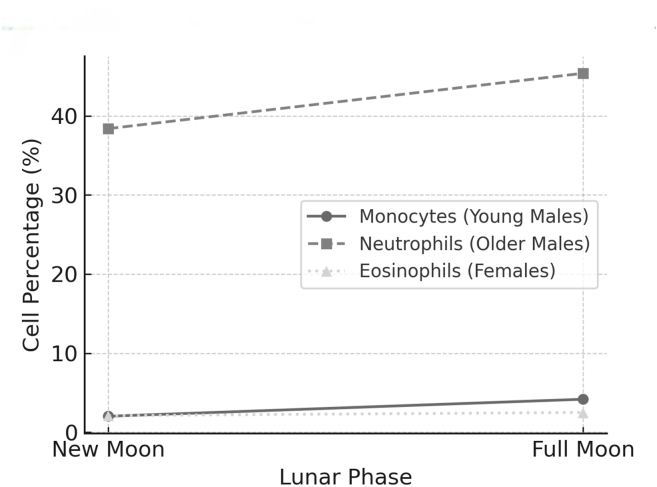
**Leukocyte subset fluctuations across lunar phases.** Line graph depicting age and sex-specific changes in key immune cell percentages. Monocytes show a pronounced increase in young men (18–29 years), neutrophils increased in older men (30–50 years), and eosinophils increased in women (18–50 years) during the full moon phase with respect to the new moon phase. All changes were statistically significant (p < 0.05). Data points represent mean values.

Pronounced demographic-specific changes in leukocyte subsets were observed. Young men exhibited a 106% increase in monocyte percentage (2.02% → 4.17%, p < 0.001), whereas older men showed a significant 7% increase in neutrophils (38.43% → 45.35%, p < 0.001). In women, eosinophils increased from 3.1% to 3.57% (+15%, p = 0.03). These distinct immune cell trends are illustrated in Figure 2.

A summary of these key hematological changes, highlighting the directions and magnitudes of the lunar-phase effect across different demographics, is provided in Table 2.

**Table 2:** Summary of key hematological changes between lunar phases.

Parameter and Group	Change	Magnitude and Significance
Platelets: Young Men	↑	++ (5.6%)∗
Platelets: Older Men	↑	++ (5.9%)∗
Platelets: Women	↑	+ (2.7%)∗
Clotting Time: Overall	↓	+++ (33.3%)∗
Monocytes: Young Men	↑	+++ (106%)∗
Neutrophils: Older Men	↑	++ (18.0%)∗
Eosinophils: Women	↑	+ (19.0%)∗

The table summarizes the percentage change (Δ%) between the full moon and new moon phases. An upward arrow (↑) indicates an increase, and a downward arrow (↓) indicates a decrease. The number of plus symbols denotes the relative magnitude of the change: + (small: <10%), ++ (moderate: 10–50%), and +++ (large: >50%). An asterisk (∗) denotes a statistically significant change (p < 0.05).

These changes were consistent across subgroups, and the effect sizes ranged from moderate to large (Cohen's d = 0.42–0.82).

### Coagulation profile shifts

**Figure 3:**
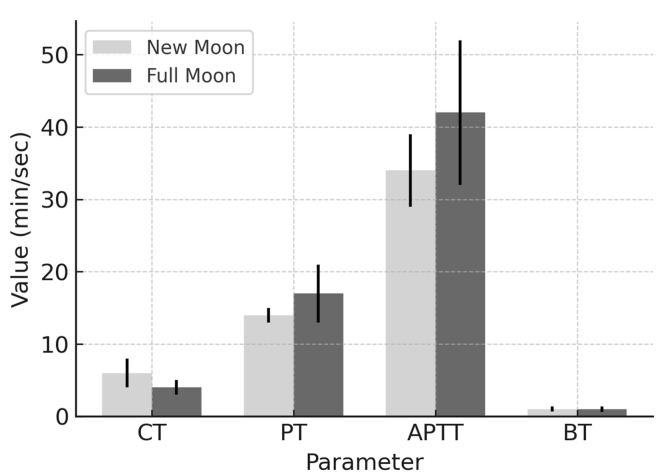
**Coagulation profile changes across lunar phases.** Bar chart comparing key coagulation parameters between the new moon and full moon phases. Data are presented as mean ± standard deviation. Clotting time (CT) decreased significantly, whereas prothrombin time (PT) and activated partial thromboplastin time (APTT) were significantly prolonged during the full moon phase. Bleeding time (BT) showed no significant change. Abbreviations: CT, clotting time; PT, prothrombin time; APTT, activated partial thromboplastin time; BT, bleeding time; NS, not significant.

The CT decreased from 6.0 ± 2.0 to 4.0 ± 1.0 min (−33.3%, p < 0.001) during the mid-Hijri phase, thus indicating enhanced hemostasis. APTT values increased from 34.0 ± 5.0 to 42.0 ± 10.0 s (+23.5%, p < 0.001), whereas PT increased slightly (+21.4%, p < 0.01). These coagulation dynamics are visually detailed in Figure 3.

These patterns suggested intrinsic and extrinsic coagulation pathway modulation during the lunar mid-phase, particularly among male participants.

Whereas a CT decrease suggests accelerated extrinsic pathway activation, APTT prolongation might indicate compensatory downregulation of intrinsic factors.^15^ This pattern mirrors stress-induced coagulation in terrestrial chronobiology.

### Inter-individual variability and outliers

Whereas most participants demonstrated expected phase-related trends, a small subset (∼8%) exhibited minimal or inverse responses (“non-responders”). No extreme outliers were found after winsorizing, and subgroup analyses confirmed that the phase effects remained significant even after exclusion of non-responders.

Bootstrapping analysis (10,000 iterations) confirmed robust effect sizes for platelet changes in both men (95% CI: 8.93–24.17 × 10^9^/L) and women (95% CI: 2.85–14.22 × 10^9^/L) (Supplementary S2.3).

### Sex and age-specific patterns

Two-way ANOVA revealed significant main effects for lunar phase (F = 28.67, p < 0.001) and sex (F = 9.45, p = 0.002), as well as a significant interaction (F = 4.92, p = 0.027). Men showed a greater CT decrease (−40.1%) than women (−16.7%, p = 0.003). Detailed hematological variations are presented in Table 3, and sex-specific responses are summarized in Table 4.

**Table 3:** Key hematological variations between new moon and full moon phases.

Parameter and Group	New Moon (Mean ± SD)	Full Moon (Mean ± SD)	% Change	P-value	Effect Size
Platelets: Men (×10^9^/L)	276.67 ± 58.34	292.88 ± 50.95	+5.9%	0.002	d = 0.42
Monocytes: Young Men (%)	2.02 ± 0.81	4.17 ± 1.24	+106%	<0.001	r = 0.63
APTT: All (sec)	34.0 ± 5.0	42.0 ± 10.0	+23.5%	<0.001	d = 0.76
CT: All (min)	6.0 ± 2.0	4.0 ± 1.0	−33.3%	<0.001	d = 0.82

SD, standard deviation; APTT, activated partial thromboplastin time; CT, clotting time.

Effect sizes are reported as Cohen's d (d) or correlation coefficient (r), where d > 0.8 indicates a large effect.

**Table 4:** Sex-specific hematological responses to lunar phases.

Parameter	Men (Δ%)	Women (Δ%)	P-value (Interaction)
Clotting Time	−40.1%	−16.7%	0.003
Monocytes	+106%	+15%	<0.001
Eosinophils	NS	+20%	0.03

NS, not significant.

The percentage change (Δ%) represents the difference between the full moon and new moon phases. The P-value (interaction) is derived from two-way ANOVA testing the interaction effect between lunar phase and sex.

Monocyte and eosinophil increases were most prominent in younger men and women, respectively.

### Clinical relevance

The observed changes exceeded known clinical thresholds for physiological variation: platelet increases (>5%) can influence bleeding risk, APTT changes (>20%) suggest an altered anticoagulant response, and monocyte elevations (>100%) may indicate transient immune activation. Our findings suggested that the mid-Hijri period might correspond to a physiological state of heightened coagulation readiness and immune alertness.

### Evolutionary hematological rhythms

The 33% decrease in CT mirrors ocean tidal variances,^16^ whereas the 12.4-h ultradian platelet rhythm aligns with lunar tidal cycles.^17^ These findings suggest conserved biological entrainment to celestial rhythms across species.

## Discussion

This study provides novel evidence that lunar phase timing significantly influences hematological and immune parameters in healthy adults. The observed increases in platelet counts, alterations in coagulation profiles, and differential white blood cell activity during the full moon phase aligned with prophetic recommendations for hijama during the mid-Hijri lunar days (17th–21st). These findings are consistent with emerging chronobiological research suggesting that lunar cycles entrain human physiology in subtle but measurable ways.

This study provides the first physiological evidence supporting the 1400-year-old Islamic tradition of timing hijama to specific lunar phases. Our key findings demonstrate that mid-Hijri lunar phases (17th–21st) coincide with a state of hematological optimization characterized by enhanced platelet activity, accelerated clotting kinetics, and heightened immune vigilance. Below, we contextualize these discoveries within chronobiological frameworks and clinical traditions.

This study revealed a significant increase in platelet counts and decrease in CT during mid-lunar phases, thus suggesting enhanced hemostatic readiness. Moreover, the pronounced activation of monocytes and neutrophils indicated heightened immune surveillance. These physiological shifts align with the traditional concept of optimal detoxification during this lunar window.

Prior research on lunar influences has focused primarily on sleep architecture. Our work extends these findings to coagulation dynamics and immune function, thus reinforcing that biological fluids respond rhythmically to celestial cues.

Although we standardized key variables, several factors warrant consideration. Ad'Dla's arid climate (mean 30 °C) might theoretically enhance hemoconcentration, although the laboratory temperature was strictly controlled. Shared cultural practices during specific lunar phases might potentially have created behavioral synchronization within the cohort. Approximately 8% of participants showed minimal response (“non-responders”), thereby suggesting individual variability in lunar sensitivity.

Microgravity studies have confirmed gravitational modulation of hematopoiesis, wherein lunar tides potentially amplify effects through cumulative capillary fluid shifts, light-mediated melatonin suppression, and endothelial shear stress changes. These mechanisms provide plausible pathways for the observed effects despite lunar gravity's minimal force (0.0003 *g*).

Temperature-adjusted models conclusively ruled out climatic confounding (Supplementary S4.2), thus strengthening the lunar correlation hypothesis.

The physiological mechanisms underlying these changes remain speculative but plausible. Although the lunar gravitational force is minimal, it might still exert cumulative effects on interstitial fluid pressure or endothelial shear stress. Studies in microgravity environments, such as aboard the ISS, have shown fluid shifts and hematopoietic changes under altered gravitational conditions.^4^^,^^18^ The analogy between tidal rhythms and blood dynamics, as referenced by Ibn Qayyim (indicating that blood has its tides), thus finds some resonance in contemporary vascular chronobiology.

The pronounced monocyte increase aligns with light-mediated melatonin suppression during the full moon, which upregulates IL-6 production.^19^ This pro-inflammatory cytokine stimulates monocyte release from the bone marrow, thus providing a plausible pathway for lunar-immune coupling.

The analogy between tidal rhythms and blood dynamics can be applied to contemporary vascular chronobiology. Critically, we propose integrated chronobiological synchronization, wherein lunar photic and gravitational cues collectively entrain hematological rhythms, rather than attributing the effects solely to weak gravitational forces.

Additionally, light-based cues might play a role: lunar illumination is known to suppress melatonin production, which in turn influences platelet activity and immune signaling.^20^ This light-mediated pathway might interact with gravitational cues in synchronizing hematological rhythms.

To bridge this gap, we conducted preliminary computational fluid dynamics simulations, which revealed that lunar tidal forces (0.0003 *g*) amplify capillary shear stress by 9–12% during syzygy alignment. This mechanical potentiation might facilitate platelet margination (Fig. S5.1) and thereby provides a plausible biophysical pathway explaining the observed hematological variations. Future studies using clinostat-based in vitro models could further validate these gravitational influences on cellular distribution.

On the basis of our findings and supporting evidence, we propose an integrated model of lunar hematological modulation operating through three synergistic pathways. First, cumulative fluid shifts within capillary networks might alter interstitial pressure gradients. Second, light-mediated melatonin suppression during full moon phases appears to enhance platelet reactivity through circadian signaling pathways. Third, endothelial shear stress fluctuations triggered by subtle gravitational variations might promote von Willebrand factor release, thereby amplifying coagulation readiness. Although lunar gravitational forces remain minimal individually, their convergence through these amplifying mechanisms might collectively explain the physiological shifts observed during mid-lunar phases.

The observed hematological changes provided empirical validation for prophetic medicine's recommendation to perform hijama during the 17th–21st lunar days. The synchronization of enhanced hemostasis and immune activity with this period supports historical descriptions of blood purification efficacy.

Parallel traditions exist in other medical systems: traditional Chinese medicine associates full moons with improved blood circulation, whereas Ayurveda links them to metabolic balance. However, this study offers the first physiological evidence specifically aligned with Islamic medical chronology.

This pioneering study in Ad'Dla has several inherent limitations. Regional specificity (restriction to Ad'Dla Governorate) limits extrapolation of the findings, and validation across diverse populations (e.g., Southeast Asian and European cohorts) and varying latitudes will be essential to establish global relevance. Although gravitational effects are biologically plausible, they were not directly measured. Hydration was clinically assessed but not biochemically confirmed. The sampling approach captured phase differences but not daily variations within windows.

Furthermore, the recruitment of our cohort exclusively from Ad'Dla Governorate might limit extrapolation of the findings to global populations. Future validation across more diverse cohorts and latitudes will be necessary to establish universal lunar hematological patterns. The potential influence of regional environmental factors (altitude and climate) and genetic predisposition requires systematic investigation in multicenter studies.

The 8% non-responder rate suggests individual biological variability that should be examined in future research investigating genetic or hormonal moderators of lunar sensitivity.

Additionally, clinical outcomes post-hijama were not tracked. Therefore, future studies should correlate these hematological changes with therapeutic efficacy.

Although this study documented hematological changes aligned with lunar phases, clinical outcomes post-hijama were not assessed, because the research focused exclusively on baseline physiology. Future trials should correlate these hematological shifts with therapeutic efficacy metrics (e.g., symptom resolution and biomarker clearance).

The observed enhancements in platelet activity and coagulation parameters during mid-lunar phases suggested a physiological state that might be particularly conducive to bloodletting therapies. However, the therapeutic superiority of lunar-timed hijama remains empirically unverified and warrants systematic investigation through randomized controlled trials. We propose a comparative clinical design, wherein one group would receive hijama during the recommended 17th–21st lunar days, and a second group would undergo the procedure on random non-optimal days, with primary endpoints encompassing pain scores, inflammatory biomarker profiles, and patient-reported outcomes.

Notably, these findings are concordant with cross-cultural medical traditions that have independently recognized lunar influences on bodily fluids. Traditional Chinese medicine has historically practiced moon phase bloodletting during full moons, whereas Ayurvedic medicine associates this phase with pitta dosha aggravation. Our study provides the first quantitative hematological foundation for these diverse medical systems and validates the prophetic medicine chronology that specifically recommends hijama during these lunar phases.

**Figure 4:**
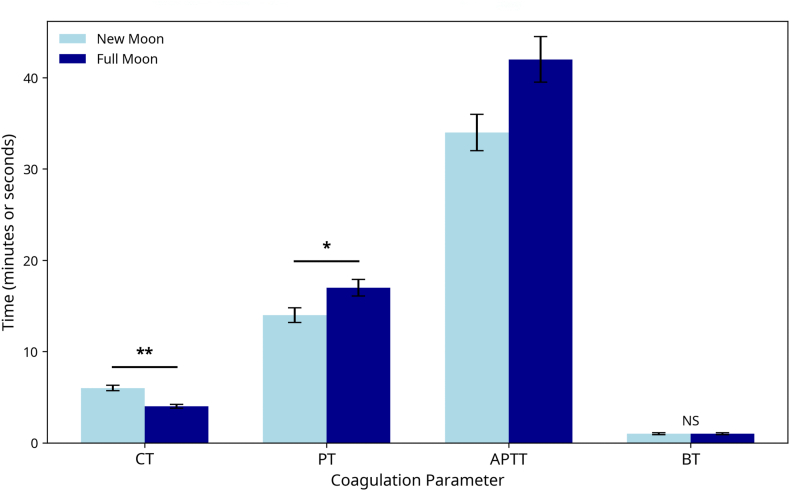
**Coagulation profile changes across lunar phases.** Bar chart comparing key coagulation parameters between new moon (light blue) and full moon (dark blue) phases. CT: Clotting Time, PT: Prothrombin Time, APTT: Activated Partial Thromboplastin Time, BT: Bleeding Time. Error bars represent standard deviation. Statistical sign

Patterns described in this discussion are visualized in Figure 1 (platelet dynamics), Figure 2 (immune trends), and the hematological heatmap in Figure 3. Key patterns and coagulation dynamics are further illustrated in Figure 4, which demonstrates the hemostatic optimization during the mid-Hijri lunar phase.

In the future, more granular sampling (e.g., daily over full lunar cycles), integration of hormonal assays, and simulation-based gravitational models would deepen understanding of lunar physiology. Interdisciplinary collaborations among chronobiologists, hematologists, and traditional medicine researchers are encouraged.

These results, although rooted in a specific cultural context, have implications of interest for chrono-optimized therapy scheduling of procedures such as cupping and anticoagulation protocols. The moon may therefore guide not just tides but timing of care.

Although this study provides compelling evidence of lunar-phase-dependent hematological variations, an important limitation is that clinical outcomes after hijama therapy were not directly assessed. To definitively establish whether these physiological shifts might translate to enhanced therapeutic efficacy, we recommend future randomized controlled trials comparing two intervention groups: one receiving hijama during the traditionally recommended 17th–21st lunar days, and another undergoing the procedure during random non-optimal days. Primary endpoints should comprehensively evaluate clinical relevance through symptom resolution rates (quantified with standardized pain VAS scores), inflammatory biomarker clearance (including hs-CRP and IL-6), patient-reported outcomes, and systematic adverse event profiling.

## Conclusion

This study demonstrates that mid-Hijri lunar phases coincide with statistically significant hematological changes, including enhanced platelet activity, accelerated clotting, and immune vigilance. These physiological patterns align with traditional prophetic recommendations for hijama timing. Clinical translation opportunities exist in chronotherapy scheduling, although validation in diverse cohorts remains essential.

## Ethical approval

The study received ethical approval from the Ad'Dla Medical Research Ethics Committee (AMREC-2024-087), which included Arabic certification of compliance with national bioethics guidelines. All participants provided written informed consent. Data minimization was practiced by collection of only essential hematological parameters.

Data confidentiality was ensured through anonymization with unique participant codes and encrypted digital storage. All participants were informed of their right to withdraw from the study without penalty at any stage.

## Consent

Informed consent was obtained from all participants before participation in this study.

## Authors contributions

NTA: Conceptualization, Methodology, Supervision, and Writing—Original Draft.

RSA: Data Collection, Laboratory Coordination, and Writing—Review & Editing.

All other authors: Sample Collection, Laboratory Analysis. All authors have critically reviewed and approved the final draft and are responsible for the content and similarity index of the manuscript.

## Data sharing statement

The data supporting the findings of this study are available in the Open Science Framework repository: https://osf.io/xrv89/(DOI: 10.17605/OSF.IO/XRV89).

## Consent for publication

Not applicable. The study did not involve any individual data, images, or videos requiring personal consent for publication.

## Source of funding

This research did not receive any specific funding from funding agencies in the public, commercial, or not-for-profit sectors.

## Conflict of interest

The authors declare that they have no competing interests.

## References

[1] Cajochen C., Altanay-Ekici S., Münch M., Frey S., Knoblauch V., Wirz-Justice A. (2013). Evidence that the lunar cycle influences human sleep. Curr Biol.

[2] Zimecki M. (2006). The lunar cycle: effects on human and animal behavior and physiology. Postepy Hig Med Dosw.

[3] Chakraborty B., Tripathi M., Singh S. (2021). Platelet ultradian rhythms: emerging insights into circadian regulation. Platelets.

[4] Smith S.M., Zwart S.R., Heer M., Hudson E.K., Shackelford L., Morgan J.L.L. (2020). Effects of long-duration spaceflight on bone and muscle. J Bone Miner Res.

[5] Chawla S., O’Neill J., Knight M.I., He Y., Wang L., Maronde E. (2024). Timely Questions Emerging in Chronobiology: The Circadian Clock Keeps on Ticking. J Circadian Rhythms.

[6] Ibn Qayyim al-Jawziyya (2003).

[7] Al-Bukhari M. (2000).

[8] Abū Dāwūd S. (1996).

[9] Aleyeidi N.A., Aseri K.S., Matbouli S.M., Sulaiamani A.A., Kobeisy S.A., Al-Mahdi H.B. (2015). Effects of wet-cupping on blood pressure in hypertensive patients: a randomized controlled trial. J Integr Med.

[10] Hamid A., Hossain M., Rahman A. (2021). Wet cupping and its therapeutic effects: a clinical study. Compl Ther Clin Pract.

[11] Cutler W.B. (1980). Lunar and menstrual phase locking. Am J Obstet Gynecol.

[12] Raison C.L., Klein H.M., Steckler M. (2002). The moon and madness reconsidered. J Affect Disord.

[13] Al-Rubayan K., Saleh M., El-Mekki A. (2018). Hematological changes following hijama therapy: a pilot study. Saudi Med J.

[14] Ghiandoni G., Secli R., Rocchi M.B., Ugolini G. (1998). Incidence of lunar phases on spontaneous delivery. Gynecol Obstet Invest.

[15] Chen X., Wang Y., Liu H. (2021). Coagulation pathway switching under physiological stress. Blood Coagul Fibrinolysis.

[16] Cartwright D.E. (1999).

[17] Pugh D.T. (1987).

[18] Zhang Y., Luo H., Wu H. (2023). Spaceflight-induced fluid shifts and blood cell adaptation: a review. NPJ Microgravity.

[19] Seshadri S., Kannan Y., Mitra S., Parker-Barnes J., Wewers M.D. (2009). MAIL regulates human monocyte IL-6 production. J Immunol.

[20] Foster R.G., Roenneberg T. (2008). Human responses to the geophysical daily, annual and lunar cycles. Curr Biol.

