# Supplementary material for: Lunar synchronization of hemostasis and immunity validates prophetic timing of hijama therapy: A multicenter study from Yemen
Source: J Taibah Univ Med Sci. 2026 Jan 6;21(1):33–40. doi: 10.1016/j.jtumed.2025.12.004 (PMC12809067; doi:10.1016/j.jtumed.2025.12.004)
Supplement: Multimedia component 1 [file mmc1.docx]

Figure S5.1. Lunar Synchronization of Platelet Activity and Immune Activation

Line graph depicting the alignment of physiological peaks (platelets, monocytes) with the 17th–21st Hijri days (shaded area). Platelet counts (red line, left axis) and white blood cell counts (green line, right axis) show synchronized increases during the full moon phase, supporting the traditional timing of hijama therapy.
