# Supplementary material for: Lunar synchronization of hemostasis and immunity validates prophetic timing of hijama therapy: A multicenter study from Yemen
Source: J Taibah Univ Med Sci. 2026 Jan 6;21(1):33–40. doi: 10.1016/j.jtumed.2025.12.004 (PMC12809067; doi:10.1016/j.jtumed.2025.12.004)
Supplement: Multimedia component 2 [file mmc2.docx]

# Supplementary S2.3 – Bootstrap Analysis (10,000 iterations)

To confirm the robustness of our findings, we performed non-parametric bootstrapping with 10,000 iterations for platelet count changes between lunar phases.

## Results

| Group | Mean Difference (×10⁹/L) | 95% Confidence Interval (Bootstrapped) |
| --- | --- | --- |
| Males | +16.21 | 8.93 – 24.17 |
| Females | +8.54 | 2.85 – 14.22 |

## Interpretation

The 95% Confidence Intervals (CIs) do not cross zero, confirming statistically significant increases in platelet counts during the full moon phase in both sexes. These results align with the paired t-test findings reported in the main manuscript.
