# Supplementary material for: Lunar synchronization of hemostasis and immunity validates prophetic timing of hijama therapy: A multicenter study from Yemen
Source: J Taibah Univ Med Sci. 2026 Jan 6;21(1):33–40. doi: 10.1016/j.jtumed.2025.12.004 (PMC12809067; doi:10.1016/j.jtumed.2025.12.004)
Supplement: Multimedia component 3 [file mmc3.docx]

Supplementary S4.2 – Temperature-Adjusted Regression Models

Given the arid climate of Ad’Dla Governorate (mean ambient temperature ≈ 30°C), we conducted sensitivity analyses to rule out temperature as a confounding variable.

Method:

We fitted multiple linear regression models with lunar phase (categorical) and ambient temperature (continuous) as predictors, and key hematological parameters as outcomes.

Key Models & Results:

| Outcome | Lunar Phase (β) | Temperature (β) | p (Phase) | p (Temp) | Adjusted R² |
| --- | --- | --- | --- | --- | --- |
| Platelet Count | +15.92 | -0.08 | 0.003 | 0.712 | 0.11 |
| CT (min) | -1.95 | +0.02 | <0.001 | 0.543 | 0.22 |
| APTT (sec) | +7.84 | -0.05 | <0.001 | 0.889 | 0.18 |

Conclusion:

Lunar phase remained a significant predictor (p < 0.01) in all models, while ambient temperature showed no significant association (p > 0.5). This confirms that the observed hematological changes are attributable to lunar phase, not climatic variation.
